# Neurology and Cognitive Occupational Therapy Practice Across the Middle East and North Africa: An Exploratory Multicountry Cross‐Sectional Survey

**DOI:** 10.1155/oti/7349715

**Published:** 2026-07-06

**Authors:** Hamad AlHamad

**Affiliations:** ^1^ Department of Occupational Therapy, Faculty of Allied Health Sciences, Health Sciences Center, Kuwait University, Kuwait City, Kuwait, kuniv.edu

**Keywords:** cognitive rehabilitation, evidence-based practice, MENA, Middle East and North Africa, neurorehabilitation, occupational therapy, stroke rehabilitation, workforce survey

## Abstract

Occupational therapy (OT) has a defined role in neurological and cognitive rehabilitation, yet empirical data describing how this role is enacted across the Middle East and North Africa (MENA) are sparse, uneven across countries, and rarely drawn from multicountry samples. In a context of rapidly rising neurological disease burden across the region, the absence of workforce‐level evidence directly constrains workforce planning, continuing‐education design, and evidence‐based practice (EBP) implementation. This exploratory cross‐sectional online survey aimed to profile occupational therapists working in neurology and cognitive rehabilitation across 13 MENA countries (using the World Bank MENA + Turkey, or “MENAT”, regional grouping). Licensed occupational therapists with at least 1 year of clinical practice were recruited between November 2023 and January 2024 through professional OT associations, networks and social media platforms. The content‐validated questionnaire was administered using Google Forms. Analyses were descriptive with Wilson 95% confidence intervals; *χ* ^2^/Fisher exact tests and Mann–Whitney *U* tests examined bivariate associations; multivariable logistic regression adjusted for key covariates; reporting followed STROBE and CHERRIES. A total of 118 occupational therapists (61.0% female; 56.8% aged 20–29 years; 78.0% bachelor‐level preparation; 58.5% in governmental services) from 13 MENA countries were analysed, with Kuwait (37.3%), Saudi Arabia (19.5%) and Jordan (11.9%) providing most responses. Stroke was the dominant clinical focus (92.4%). Neurodevelopmental treatment (71.2%, 95% CI 62.4–78.6), biomechanical (65.3%) and motor‐control/motor‐learning (63.6%) were the most commonly reported frames of reference; the Functional Independence Measure (65.3%) and Montreal Cognitive Assessment (61.9%) were the most commonly reported standardised tools. Perceived EBP challenges were reported by 72.0% of respondents. Stratified analyses revealed that postgraduate‐qualified respondents reported more organisational barriers than bachelor‐level colleagues (73.1% vs. 45.7%, *p* = 0.025), private‐sector respondents reported more distinct barriers overall (Cohen *d* = 0.54, *p* = 0.005), and respondents practising outside the Gulf Cooperation Council (GCC) subregion reported substantially higher rates of confidence‐related and evidence‐quality barriers (all *p* ≤ 0.008). Conference attendance was independently associated with use of motor‐learning approaches (adjusted OR 3.72, 95% CI 1.44–9.58; *p* = 0.007). Findings identify an evidence‐practice gap in the predominance of neurodevelopmental treatment and highlight structural, subregion‐linked barriers to EBP across the MENA region. Strengthening organisational support, protecting time for evidence use, expanding postgraduate neuro‐OT pathways, and coordinating regional workforce research and assessment‐adaptation are priorities aligned with the WHO Rehabilitation 2030 workforce agenda.

## 1. Introduction

Occupational therapy (OT) plays a central role in neurological and cognitive rehabilitation by addressing the consequences of neurological disease for occupational performance, participation and quality of life [1, 2]. People living with stroke, traumatic brain injury, multiple sclerosis, Parkinson disease, dementia and related conditions commonly experience limitations across self‐care, productivity, leisure, cognition and social engagement. Within integrated neurorehabilitation, occupational therapists contribute assessment of occupational performance and neurocognitive function, enablement of adaptation and promotion of meaningful engagement in daily life [3, 4]. Contemporary neurological rehabilitation emphasises person‐centred, occupation‐focused and interdisciplinary approaches that align closely with the core philosophy of OT [1, 5].

The burden of neurological conditions across the Middle East and North Africa (MENA) is substantial and rising. Recent Global Burden of Disease analyses report that disability‐adjusted life‐years (DALYs) attributable to neurological disorders in MENA approximately doubled between 1990 and 2019, driven largely by dementias, stroke and movement disorders in an ageing, urbanising population [6, 7]. Cieza and colleagues estimated that 2.41 billion people globally required rehabilitation in 2019, a 63% rise since 1990, with neurological conditions contributing a disproportionate share of the unmet need [8]. This rising demand has placed OT services—often small, unevenly regulated and concentrated in tertiary‐hospital contexts across the region—under increasing strain.

Although OT in neurology is well established in many high‐income settings, the organisation, visibility, educational pathways and regulatory frameworks of the profession vary considerably across MENA countries. Existing regional work has documented variation in service organisation, interprofessional awareness, professional recognition and the implementation of evidence‐based practice (EBP), as well as ongoing needs for workforce development [9–16]. Jesus et al.′s [17] recent scoping review of the global OT workforce research base found that only a small proportion of the evidence originates from resource‐limited or underrepresented regions, with MENA conspicuously underrepresented. OT practice in the region may also be shaped by culture, language, family involvement, religious values and health‐system organisation; these have been discussed conceptually but rarely measured empirically across multiple countries [18–21].

Despite the growing burden of neurological disease and the increasing visibility of OT in the region, remarkably little empirical evidence describes how neurology and cognitive OT is actually practised across MENA countries. Published regional studies have tended to focus on single countries [9, 11, 12, 14, 15], single diagnostic groups, or broader professional issues rather than multicountry descriptions of neurology/cognition‐focused practice. A clearer understanding of who delivers this care, in what settings, using which models, assessments and approaches, and against which organisational barriers is important because such descriptive data enable workforce planning, identify priority targets for continuing professional development and generate hypotheses for implementation research. These priorities are directly aligned with the WHO Rehabilitation 2030 initiative and with the 2023 World Health Assembly resolution WHA76.6 on strengthening rehabilitation in health systems [22, 23].

The aim of this study was to describe, in an exploratory cross‐sectional design, the demographic and professional characteristics, practice settings, reported models and frames of reference (FoR), assessment tools, occupational areas targeted in intervention and perceived EBP barriers among occupational therapists working in neurology and cognitive rehabilitation in a convenience sample across 13 MENA countries. Rather than seeking representative regional estimates, the study was designed to provide an initial descriptive snapshot and to identify directions for country‐specific and multicentre research, for curriculum and continuing‐education reform and for service and policy development.

## 2. Materials and Methods

### 2.1. Study Design and Reporting

This exploratory cross‐sectional online survey followed the Strengthening the Reporting of Observational Studies in Epidemiology (STROBE) statement for cross‐sectional studies [24] and the Checklist for Reporting Results of Internet E‐Surveys (CHERRIES) [25].

### 2.2. Regional Definition and Eligibility

The MENA is a recognised regional grouping in the international health, workforce and burden‐of‐disease literature, although different definitions are used across contexts [26]. For the purpose of this study, the region was operationally defined using the World Bank MENA classification [27] augmented with Turkey—a grouping sometimes denoted “MENAT” in regional health‐workforce work—to encompass the 13 countries from which respondents were ultimately drawn: Kuwait, Saudi Arabia, the United Arab Emirates, Qatar, Bahrain, Oman, Jordan, Palestine, Lebanon, Syria, Morocco, Iran and Turkey. This definition was selected for three connected reasons. First, it aligns with the regional groupings used in the principal burden‐of‐disease and rehabilitation‐need literature relevant to this study, including the Global Burden of Disease MENA analyses [6, 7] and the WHO Eastern Mediterranean Region (EMRO) workforce reports [28] within whose remit most of these countries lie. Second, it corresponds to the practical reach of regional professional networks: the Arab OT Regional Group (AOTRG), a WFOT regional member since 2012 [29], explicitly bridges the Arabian Peninsula, the Levant and North Africa and Iran and Turkey share long‐standing professional and educational connections with Levantine and Arabian Peninsula OT communities. Third, restricting the sample to a narrower ‘Middle East‐only’ definition would force the exclusion of substantively important regional contexts (notably Morocco) for which the rehabilitation‐need profile and OT workforce challenges are documented as broadly congruent with those of the wider MENA region [9, 17, 30]. We acknowledge that other regional definitions are used in the literature (e.g., narrower West Asia/Arabian Peninsula groupings, broader EMRO with 22 countries, or the GCC [Gulf Cooperation Council] alone) and that any regional classification involves analytic choices; we therefore report results stratified by subregion (GCC vs. non‐GCC MENA) so that readers using narrower definitions can interpret the findings against the most relevant subset of the sample.

**Figure 1:**
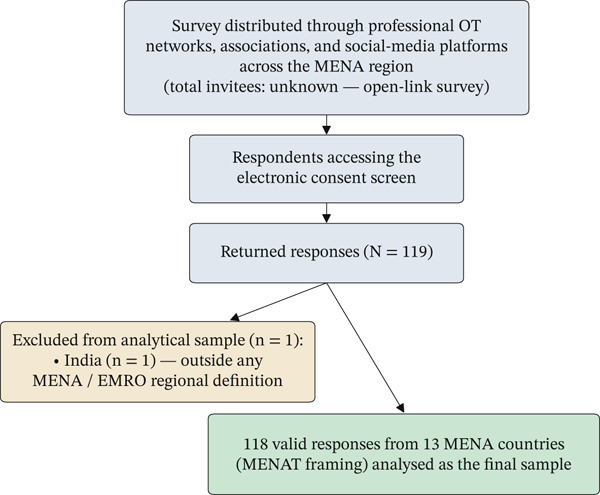
Participant flow diagram. After exclusion of one out‐of‐region respondent (India), 118 valid responses from 13 MENA countries formed the analytical sample. Reporting follows the Checklist for Reporting Results of Internet E‐Surveys (CHERRIES).

Eligible participants were licensed occupational therapists with at least 1 year of clinical practice experience who were currently working in one of the 13 listed countries. Participants were required to self‐identify as active in adult neurological or cognitive rehabilitation practice. Because the survey was advertised through open professional and social media channels, one respondent did not meet the regional eligibility criterion post hoc—a respondent practising in India, which lies outside any conventional MENA or EMRO definition. This response was excluded from the analytical sample; the exclusion is documented transparently in the participant flow diagram (Figure 1) and a sensitivity analysis confirmed that retaining versus excluding this single response did not materially alter any reported descriptive findings or inferential directions. Incomplete or invalid responses (none identified) were also excluded prior to analysis.

### 2.3. Recruitment

Recruitment used convenience and snowball sampling [31]. Invitations were disseminated through national and regional OT associations, professional contacts and social media platforms (Facebook, LinkedIn, WhatsApp); recipients were encouraged to share the invitation with eligible colleagues. Because the link was distributed through open online channels, the total number of occupational therapists who received the invitation could not be determined, and a conventional response rate could not be calculated; the completion rate (fully completed/started) is reported instead, consistent with CHERRIES guidance [25]. The survey was open from 7 November 2023 to 7 January 2024.

### 2.4. Survey Instrument, Pretesting and Acknowledged Limitations

A structured, self‐report questionnaire was developed specifically for this study to capture the practice profile of neurology and cognitive OT. The instrument included five domains: (1) demographic and professional characteristics; (2) practice context (work sector, interdisciplinary team frequency, service setting, caseload and primary client age group); (3) clinical populations; (4) reported practice approaches (OT models, FoR, assessment tools and reasons for selection); and (5) intervention focus and EBP. Most items were categorical with single‐ or multiple‐response options; free‐text fields allowed respondents to add tools or comments not listed.

Content validity was assessed using the Polit and Beck procedure [32, 33]. Five content experts with combined expertise in OT, neurology, rehabilitation, education and health‐services research reviewed each item for relevance on a four‐point scale. Item‐level content validity index (I‐CVI) values ranged across items, and the scale‐level content validity index (S‐CVI/Ave) was acceptable against the recommended threshold of ≥ 0.90 [33]. Following content‐validity review, the instrument was pilot‐tested with 30 occupational therapists to assess clarity, face validity, completion time (mean ≈ 12 min) and usability across languages; minor wording and response‐format revisions were made before deployment.

Three instrument limitations identified during data auditing are acknowledged here for transparency and are addressed in Section 5. First, the deployed form contained a minor typographical error (‘client‐cantered’ for client‐centered) and ambiguous phrasing in one of the orientation screens (‘translated to resistance country language’ was the draft phrasing for resident country/local language); these wording errors did not alter response options and, in the author′s assessment, are unlikely to have systematically biased responses, but they are disclosed for full transparency. Second, the monthly‐income categories as deployed (‘≥ 1000’, ‘1100–2100’, ‘2200–3000’, ‘≤ 3100’) contain logical inconsistencies in their boundary markers that prevent ordered analysis; income is therefore reported descriptively only and is not used in any inferential model. Third, the draft appendix listed marital‐status options as ‘Married /Not married’, whereas the deployed form provided three distinct options (Single/Married/Divorced) and all analyses reflect the three‐option format from the deployed form; no recoding or collapsing was performed. The draft appendix additionally listed ‘OT assistant’ as a qualification option, but this option was not selected by any respondent and is not relevant to the eligibility criterion (licensed occupational therapist). Given the descriptive and predominantly multiple‐response nature of the instrument, formal psychometric testing of total scores was not undertaken, and a reliability analysis of the six‐item EBP‐barrier inventory is reported in Section 3.5.

### 2.5. Data Collection and Ethics

The questionnaire was administered online using Google Forms. Before accessing the questionnaire, participants were presented with an electronic participant information sheet and consent statement outlining the study purpose, voluntariness, anonymity, confidentiality and the right to withdraw prior to submission. No directly identifying information was collected, except for an optional contact field for respondents willing to be approached for a subsequent qualitative interview. A duplicate‐prevention setting restricted one response per Google account; no incentives were offered. The questionnaire displayed a progress indicator, used forward and backward navigation and permitted review before submission. The study was approved by the Kuwait University Ethical Review Board (Approval No. 312) and adhered to the Declaration of Helsinki.

### 2.6. Statistical Analysis Plan

All analyses were planned a priori and were conducted using SPSS Version 31. Sample size was determined pragmatically; no a priori power calculation was performed, consistent with the exploratory descriptive aim. For a sample of *N* = 118 and an observed proportion of 0.50, the precision‐based (Cochran) 95% margin of error for point estimates is approximately ±9 percentage points [34], providing adequate precision for descriptive workforce profiling and sensitivity to medium‐to‐large bivariate associations (Cohen *w* ≈ 0.30) but limited power for small effects [35]. Effect sizes and confidence intervals are therefore reported throughout.

Descriptive statistics comprised frequencies and Wilson score 95% confidence intervals [36] for categorical variables and means, standard deviations, medians and interquartile ranges (IQRs) for continuous or pseudocontinuous variables. For multiple‐response items, the denominator was the total analytical sample (*N* = 118); percentages within domains may therefore sum to more than 100%.

Bivariate associations were examined using the *χ* ^2^ test or Fisher′s exact test when any expected cell count was below five [35]; the Mann–Whitney *U* test or Kruskal–Wallis test was used for ordinal or non‐normal continuous outcomes. Effect sizes were reported as *φ* or Cramér′s *V* for categorical associations and as the rank‐biserial correlation *r* = |*z*|/√*N* for rank‐based tests. For ordinal predictors, Spearman rank correlations (*ρ*) were calculated. Bivariate findings guided the selection of covariates for multivariable models.

Multivariable logistic regression was used to estimate adjusted odds ratios (aORs) with 95% confidence intervals for three pre‐specified outcomes: (i) reporting perceived EBP challenges (yes/no), (ii) high frequency of EBP use (≥ ‘most of the time’) and (iii) reported use of motor‐control/motor‐learning FoR. Covariates were work sector (private vs. governmental), years of practice (midpoint of reported bands), subregion (GCC countries vs. other MENA), conference attendance, highest qualification (postgraduate vs. bachelor) and gender.

The analyses described above (descriptive profile, three prespecified multivariable models, internal‐consistency analysis) were planned a priori. In addition, a series of post hoc, exploratory stratified bivariate analyses of the six perceived EBP barriers was added in response to peer‐review input, separately by (a) years of practice (three‐band grouping: 1–5, 6–15, ≥ 16 years), (b) work sector (governmental vs. private), (c) highest qualification (bachelor vs. postgraduate) and (d) subregion (GCC vs. other MENA). These post hoc subgroup analyses were not adjusted for multiple comparisons and several subgroup cells are small; consequently, they are reported as exploratory signals that require confirmation in larger, country‐specific studies rather than as robust subgroup effects. These stratified analyses are reported in Table 1 and support the interpretive discussion of the barrier profile (Section 4.3). A four‐way subregional breakdown (GCC, Levant, Maghreb [Morocco], non‐Arab MENAT [Iran/Turkey]) is reported descriptively for the most strongly differentiated barriers but is not subjected to formal inference because of small cell sizes in the Maghreb and non‐Arab MENAT strata.

**Table 1:** Perceived EBP barriers, stratified by years of practice, work sector, highest qualification and subregion (*N* = 118). Values are percentage reporting each barrier with bivariate test *p* values; significant cells (*p* < 0.05) are bolded.

EBP barrier	1–5y vs. 6–15y vs. ≥ 16y	Gov vs. priv	Bachelor vs. postgrad	GCC vs non‐GCC
Organisational support	43.8/63.3/58.3 *p* = 0.160	49.3 vs. 55.1 *p* = 0.578	**45.7 vs. 73.1 *p* = 0.025**	49.4 vs. 56.8 *p* = 0.586
Time to search evidence	42.2/46.7/66.7 *p* = 0.121	42.0 vs. 57.1 *p* = 0.135	43.5 vs. 65.4 *p* = 0.080	45.7 vs. 54.1 *p* = 0.518
Time to incorporate evidence	39.1/50.0/50.0 *p* = 0.491	40.6 vs. 49.0 *p* = 0.452	43.5 vs. 46.2 *p* = 0.985	40.7 vs. 51.4 *p* = 0.380
Poor evidence quality	34.4/43.3/25.0 *p* = 0.371	29.0 vs. 42.9 *p* = 0.169	34.8 vs. 34.6 *p* = 1.000	**25.9 vs. 54.1 *p* = 0.006**
Low confidence in appraising	20.3/13.3/29.2 *p* = 0.356	**11.6 vs. 32.7 *p* = 0.010**	19.6 vs. 23.1 *p* = 0.907	**12.3 vs. 37.8 *p* = 0.003**
Low confidence in implementing	12.5/23.3/33.3 *p* = 0.074	14.5 vs. 26.5 *p* = 0.156	17.4 vs. 26.9 *p* = 0.422	**12.3 vs. 35.1 *p* = 0.008**
Number of barriers (mean ± SD)	1.95 ± 1.18/2.40 ± 1.40/2.62 ± 2.06 *p* = 0.244 (KW)	**1.87 ± 1.33 vs. 2.63 ± 1.55 *p* = 0.005 (MW); d = 0.54**	2.07 vs. 2.81 *p* = 0.080 (MW)	**2.02 vs. 2.59 *p* = 0.046 (MW)**

*Note:* Cells, % reporting the barrier (or mean ± SD for count). Tests, *χ* ^2^ (Fisher when expected < 5); Bolded cells indicate *p* < 0.05. Descriptive 4‐way breakdown for the most strongly differentiated barriers (GCC/Levant/Maghreb/non‐Arab MENAT). Confidence appraising 12.3%/29.2%/25.0%/100.0%. Confidence implementing 12.3%/29.2%/12.5%/100.0%. Poor evidence quality 25.9%/54.2%/37.5%/80.0%. Maghreb (*n* = 8) and non‐Arab MENAT (*n* = 5) cells are descriptive only and are not subjected to formal inference. Non‐GCC subregion comprises Levant, Maghreb (Morocco) and non‐Arab MENAT (Iran, Turkey).

Abbreviations: KW, Kruskal–Wallis; MW, Mann–Whitney. Postgrad, Master/PhD/OTD.

Internal consistency of the six‐item EBP‐barriers inventory was examined using Cronbach *α* and item–total correlations, both in the full sample and among the subsample reporting at least one barrier. All tests were two‐sided with *α* = 0.05; given the exploratory and hypothesis‐generating nature of the analyses, no formal adjustment for multiple testing was applied and inferential findings are interpreted as hypothesis‐generating signals requiring confirmation in future country‐specific and multicentre research.

## 3. Results

### 3.1. Respondent Characteristics

Between 7 November 2023 and 7 January 2024, 119 responses were received. Following exclusion of one out‐of‐region respondent (India; see Figure 1), 118 valid responses from 13 MENA countries formed the analytical sample. Detailed demographic and professional characteristics are presented in Table 2. The sample was predominantly female (72/118, 61.0%; 95% CI 52.0–69.3) and early‐career, with 56.8% (67/118) aged 20–29 years and 54.2% (64/118) reporting 1–5 years of OT experience. Most respondents held a bachelor‐level qualification as their highest degree (92/118, 78.0%), with master′s (13.6%), PhD (5.1%) and OTD (3.4%) representing minority pathways.

**Table 2:** Demographic and professional characteristics of the analytical sample (*N* = 118).

Variable/category	*n*	%	95% CI
Gender			
Female	72	61.0	52.0–69.3
Male	46	39.0	30.7–48.0
Age group (years)			
20–29	67	56.8	47.8–65.4
30–39	28	23.7	17.0–32.2
40–49	18	15.3	9.9–22.8
50–59	3	2.5	0.9–7.2
≥ 60	2	1.7	0.5–6.0
Marital status			
Single	62	52.5	43.6–61.3
Married	53	44.9	36.2–53.9
Divorced	3	2.5	0.9–7.2
Highest qualification			
Bachelor	92	78.0	69.7–84.5
Master	16	13.6	8.5–20.9
PhD	6	5.1	2.4–10.7
OTD	4	3.4	1.3–8.4
Years practicing as an OT			
1–5	64	54.2	45.3–63.0
6–10	19	16.1	10.6–23.8
11–15	11	9.3	5.3–15.9
16–20	12	10.2	5.9–16.9
> 20	12	10.2	5.9–16.9
Country of practice			
Kuwait	44	37.3	29.1–46.3
Saudi Arabia	23	19.5	13.4–27.6
Jordan	14	11.9	7.2–18.9
Morocco	8	6.8	3.5–12.8
United Arab Emirates	7	5.9	2.9–11.7
Palestine	6	5.1	2.4–10.7
Qatar	4	3.4	1.3–8.4
Iran	3	2.5	0.9–7.2
Bahrain, Lebanon, Syria, Turkey (each)	2	1.7	0.5–6.0
Oman	1	0.8	0.1–4.6
Subregion			
GCC (KW, KSA, UAE, Qatar, Bahrain, Oman)	81	68.6	59.8–76.3
Levant (Jordan, Palestine, Lebanon, Syria)	24	20.3	14.1–28.5
Maghreb (Morocco)	8	6.8	3.5–12.8
Non‐Arab MENAT (Iran, Turkey)	5	4.2	1.8–9.5
Work sector			
Governmental	69	58.5	49.5–67.0
Private	49	41.5	33.0–50.5
Daily caseload			
1–3	44	37.3	29.1–46.3
4–7	61	51.7	42.8–60.5
> 7	13	11.0	6.6–17.9
Interdisciplinary teamwork frequency			
Every week	36	30.5	22.9–39.3
Every month	20	16.9	11.2–24.7
Every 2 weeks	4	3.4	1.3–8.4
Once every 2 months or less	26	22.0	15.5–30.3
Never	32	27.1	19.9–35.8
Primary client age group			
Adult (19–64)	95	80.5	72.4–86.6
Adolescent (13–18)	14	11.9	7.2–18.9
Geriatric (≥ 65)	9	7.6	4.1–13.9
Attended a neurology/cognition conference			
Yes	91	77.1	68.8–83.8
No	27	22.9	16.2–31.2

*Note:* Percentages within category‐groups sum to 100 ± rounding. Wilson score 95% confidence intervals are reported. Monthly income is not tabulated due to inconsistent interval labels in the deployed form (see Methods Sections 2.4 and 5).

Abbreviation: MENAT, Middle East and North Africa + Turkey.

Kuwait (37.3%), Saudi Arabia (19.5%) and Jordan (11.9%) contributed the three largest country subsamples and together represented 68.6% of the analytical sample; Morocco (6.8%) and the United Arab Emirates (5.9%) followed. Subregional composition was as follows: GCC 68.6% (*n* = 81), Levant 20.3% (*n* = 24), Maghreb (Morocco) 6.8% (*n* = 8) and non‐Arab MENAT (Iran + Turkey) 4.2% (*n* = 5). A majority of respondents reported working in governmental services (69/118, 58.5%), with the remainder in the private sector (41.5%). Daily caseload was 4–7 patients for the largest group (51.7%); 30.5% reported weekly interdisciplinary team engagement, whereas 27.1% reported never engaging with an interdisciplinary team. Adult clients (aged 19–64 years) represented the primary caseload focus for 80.5% of respondents.

### 3.2. Clinical Populations and Practice Settings

Stroke dominated the neurological caseload, with 92.4% (109/118) of respondents naming it among their three most treated conditions. Traumatic and acquired brain injury (68.6%), spinal cord injury (47.5%), multiple sclerosis (31.4%), peripheral neuropathy (27.1%), dementia (20.3%), Parkinson disease (16.9%) and psychiatric conditions (0.8%) followed. Detailed counts and confidence intervals appear in Table 3. Interdisciplinary‐team frequency correlated moderately with the number of neurological diagnoses treated (Spearman *ρ* = 0.38, *p* < 0.001) and strongly with the number of standardised assessments used (*ρ* = 0.51, *p* < 0.001), suggesting that team‐based practice clusters with broader clinical exposure and more standardised assessment behaviour.

**Table 3:** Neurological conditions most commonly treated by respondents (*N* = 118). Each respondent selected up to three diagnostic groups from a fixed list; the table summarises the proportion of respondents naming each condition among their top three.

Clinical focus	*n*	%	95% CI
Stroke	109	92.4	86.1–95.9
Traumatic/acquired brain injury	81	68.6	59.8–76.3
Spinal cord injury	56	47.5	38.7–56.4
Multiple sclerosis	37	31.4	23.7–40.2
Peripheral neuropathy	32	27.1	19.9–35.8
Dementia/Alzheimer disease	24	20.3	14.1–28.5
Parkinson disease	20	16.9	11.2–24.7
Psychiatric conditions	1	0.8	0.1–4.6

*Note:* Respondents identified their three most commonly treated diagnostic groups; percentages may sum to more than 300%.

### 3.3. Reported Models and FoR

Among OT models, the Person–Environment–Occupation (PEO) model (67/118, 56.8%) and the Canadian Model of Occupational Performance and Engagement (CMOP‐E; 65/118, 55.1%) were reported most frequently, followed by the Model of Human Occupation (MOHO; 48.3%) and Occupational Adaptation (OA; 33.1%); 95.0% of respondents endorsed at least one recognised occupation‐based model. Use of PEO varied across years‐of‐practice bands (*χ* ^2^(4) = 9.50, *p* = 0.050)

**Figure 2:**
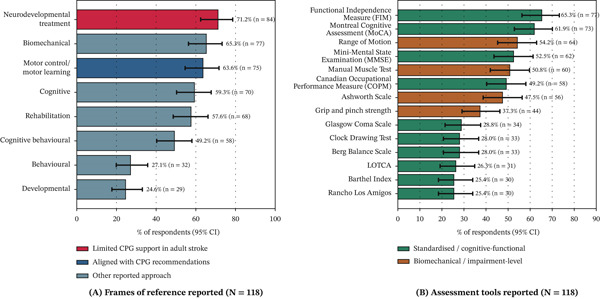
Reported practice approaches (*N* = 118). (A) Frames of reference, with Wilson 95% CIs. (B) Assessment tools, colour‐coded as standardised/cognitive‐functional or biomechanical/impairment‐level.

FoR used in neurology and cognition are summarised in Table 4 and Figure 2A. Neurodevelopmental treatment (NDT/Bobath) was the most commonly reported approach (84/118, 71.2%, 95% CI 62.4–78.6), followed by the biomechanical FoR (65.3%), motor‐control/motor‐learning (63.6%), cognitive (59.3%), rehabilitation (57.6%), cognitive‐behavioural (49.2%), behavioural (27.1%) and developmental (24.6%) approaches. Reported NDT use was higher among female than male respondents (79.2% vs. 58.7%; *χ* ^2^(1) = 4.78, *p* = 0.029; OR 2.67, 95% CI 1.18–6.06). Conference attendance was strongly associated with use of motor‐control/motor‐learning approaches (unadjusted OR 4.16, 95% CI 1.66–10.41, *p* = 0.002; adjusted OR 3.72, 95% CI 1.44–9.58, *p* = 0.007, controlling for sector, qualification, subregion and years of practice).

**Table 4:** Reported OT models, frames of reference, assessment tools and occupational areas targeted in neurology and cognitive rehabilitation practice (*N* = 118; multiple responses permitted within each domain).

Domain and item	*n*	%	95% CI
**Occupational therapy models**			
Person–Environment–Occupation (PEO)	67	56.8	47.8–65.4
Canadian Model of Occupational Performance and Engagement (CMOP‐E)	65	55.1	46.1–63.8
Model of Human Occupation (MOHO)	57	48.3	39.5–57.2
Occupational Adaptation (OA)	39	33.1	25.2–42.0
**Frames of reference/practice approaches**			
Neurodevelopmental treatment (NDT/Bobath)	84	71.2	62.4–78.6
Biomechanical	77	65.3	56.3–73.2
Motor control/motor learning	75	63.6	54.6–71.7
Cognitive	70	59.3	50.3–67.8
Rehabilitation	68	57.6	48.6–66.2
Cognitive behavioural	58	49.2	40.3–58.1
Behavioural	32	27.1	19.9–35.8
Developmental	29	24.6	17.7–33.1
**Assessment tools (most commonly reported)**			
Functional Independence Measure (FIM)	77	65.3	56.3–73.2
Montreal Cognitive Assessment (MoCA)	73	61.9	52.9–70.1
Range of Motion	64	54.2	45.3–63.0
Mini‐Mental State Examination (MMSE, any variant)	62	52.5	43.6–61.3
Manual Muscle Test	60	50.8	41.9–59.7
Canadian Occupational Performance Measure (COPM)	58	49.2	40.3–58.1
Ashworth Scale	56	47.5	38.7–56.4
Grip and pinch strength	44	37.3	29.1–46.3
Glasgow Coma Scale (GCS)	34	28.8	21.4–37.6
Clock Drawing Test	33	28.0	20.7–36.7
Berg Balance Scale	33	28.0	20.7–36.7
Loewenstein OT Cognitive Assessment (LOTCA)	31	26.3	19.2–34.9
Barthel Index	30	25.4	18.4–34.0
Rancho Los Amigos scale	30	25.4	18.4–34.0
**Occupational areas targeted in intervention**			
Activities of daily living (ADL)	114	96.6	91.6–98.7
Social participation	76	64.4	55.4–72.5
Work	71	60.2	51.2–68.5
Instrumental ADL (IADL)	69	58.5	49.5–67.0
Education	60	50.8	41.9–59.7
Leisure	59	50.0	41.1–58.9
Health management	48	40.7	32.2–49.7
Play	48	40.7	32.2–49.7
Rest and sleep	45	38.1	29.9–47.1

*Note:* Multiple responses were permitted within each domain. 95% CI is Wilson score 95% confidence interval.

Reasons reported for model or frame‐of‐reference selection were dominated by therapist′s clinical reasoning (82.2%) and area of practice (65.3%), followed by reasons for referral (36.4%), client′s age group (35.6%) and organisational rules (15.3%).

### 3.4. Assessment Tools and Occupational Areas Targeted

Reported assessment tools are summarised in Table 4 and Figure 2B. The Functional Independence Measure (FIM; 65.3%) and Montreal Cognitive Assessment (MoCA; 61.9%) were the two most commonly reported standardised tools, followed by Range of Motion assessment (54.2%), the Mini‐Mental State Examination (52.5%), Manual Muscle Test (50.8%), the Canadian Occupational Performance Measure (COPM; 49.2%), Ashworth Scale (47.5%), grip and pinch strength (37.3%), Glasgow Coma Scale (28.8%), Clock Drawing Test and Berg Balance Scale (each 28.0%), Loewenstein OT Cognitive Assessment (LOTCA; 26.3%) and Rancho Los Amigos scale and Barthel Index (each 25.4%). The most reported drivers of assessment selection were availability at the workplace, training at university and perceived clinical utility.

Areas of occupation targeted in intervention (Table 4) were led by activities of daily living (ADL; 96.6%), social participation (64.4%), work (60.2%), instrumental ADL (58.5%), education (50.8%), leisure (50.0%), play and health management (each 40.7%) and rest/sleep (38.1%). Respondents addressed a median of 5 occupational areas (IQR 3–7; mean 5.00, SD 2.55).

### 3.5. EBP Frequency, Barriers and Reliability

**Figure 3:**
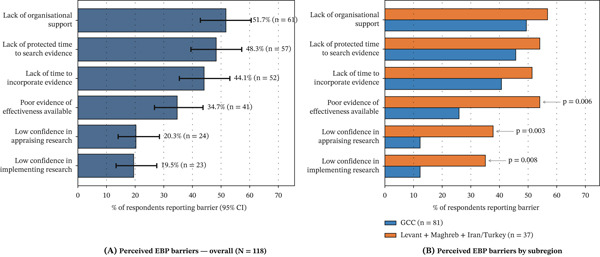
Perceived EBP barriers (*N* = 118). (A) Overall proportions with Wilson 95% CIs. (B) proportions by GCC versus non‐GCC subregion (Levant + Maghreb + Iran/Turkey). Subregional differences were significant for confidence appraising (*p* = 0.003), confidence implementing (*p* = 0.008) and poor evidence quality (*p* = 0.006).

Frequency of EBP use was reported as ‘Always’ by 16.9%, ‘Most of the time’ by 43.2%, ‘Sometimes’ by 30.5%, ‘Less often’ by 5.9% and ‘Never’ by 3.4% of respondents. Perceived EBP challenges were reported by 72.0% (85/118) of respondents. The six specific barriers are displayed overall and by subregion in Figure 3. In the full sample, the most frequently reported were lack of organisational support (51.7%, 95% CI 42.8–60.5), lack of protected time to search evidence (48.3%), lack of time to incorporate evidence (44.1%), availability of poor‐quality evidence (34.7%), low confidence in appraising research (20.3%) and low confidence in implementing research findings (19.5%)

**Figure 4:**
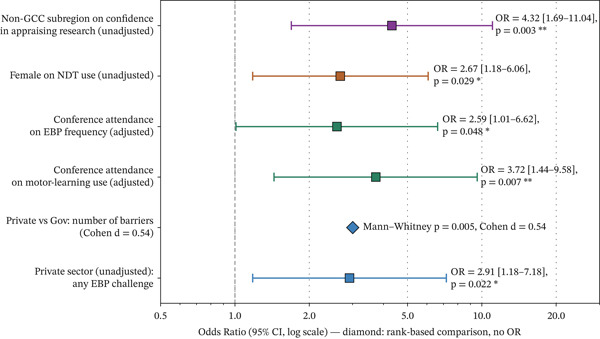
Forest plot of key associations (*N* = 118). Odds ratios (95% CI, log scale) for binary outcomes; the diamond marker summarises the rank‐based comparison of barrier counts by sector (Cohen *d* = 0.54).

Stratified bivariate comparisons for each barrier are reported in full in Table 1. Three patterns warrant particular attention. (a) Postgraduate‐qualified respondents (*n* = 26) endorsed the organisational‐support barrier significantly more often than bachelor‐level colleagues (73.1% vs. 45.7%; *χ* ^2^(1) = 5.04, *p* = 0.025), with a similar nonsignificant trend for time to search (65.4% vs. 43.5%, *p* = 0.080). This counter‐intuitive ‘more training, more barriers reported’ pattern is discussed in Section 4.3. (b) Work‐sector differences were concentrated in a single item: low confidence in appraising research was reported by 32.7% of private‐sector respondents compared with 11.6% of governmental‐sector respondents (*χ* ^2^(1) = 6.60, *p* = 0.010; Fisher *p* = 0.010), and the number of distinct barriers reported was substantially higher in the private sector (mean 2.63, SD 1.55) than the governmental sector (mean 1.87, SD 1.33; Mann–Whitney *U* = 1186, *p* = 0.005; Cohen *d* = 0.54, medium effect). The unadjusted private‐versus‐governmental odds ratio for any reported EBP challenge was 2.91 (95% CI 1.18–7.18; Fisher *p* = 0.022); these key bivariate associations, together with the Mann–Whitney comparison of barrier counts, are summarised in the forest plot (Figure 4). (c) Subregional differences were larger than those for any other stratifier, although they should be read with the multiplicity and small‐cell caveats noted above: respondents practising outside the GCC (Levantine countries, Morocco, Iran and Turkey; combined *n* = 37) reported much higher rates of the three evidence‐quality and confidence‐related barriers than GCC respondents—poor evidence quality 54.1% versus 25.9% (*χ* ^2^ = 7.51, *p* = 0.006), low confidence in appraising research 37.8% versus 12.3% (*p* = 0.003; OR 4.32, 95% CI 1.69–11.04) and low confidence in implementing research findings 35.1% versus 12.3% (*p* = 0.008). Organisational and time‐related barriers did not differ significantly by GCC vs non‐GCC subregion.

A descriptive four‐way subregional breakdown (Table 1 footnote) showed the highest barrier rates among the small non‐Arab MENAT subsample (Iran + Turkey, *n* = 5), followed by Levantine respondents, with the GCC subsample reporting the lowest rates across confidence‐related items. The Maghreb (*n* = 8) and non‐Arab MENAT (*n* = 5) subsamples are too small for stable inference, and the broader non‐GCC grouping aggregates culturally, linguistically, economically, politically and professionally heterogeneous contexts; the four‐way pattern is therefore reported descriptively only. It is consistent with, rather than independent of, the binary GCC versus non‐GCC analysis above; both should be read as exploratory signals warranting targeted country‐specific follow‐up rather than as definitive subregional effects.

In the primary adjusted multivariable model for any EBP challenge (Table 5), the work‐sector signal attenuated to borderline (aOR 2.51, 95% CI 0.90–7.01, *p* = 0.078) after accounting for subregion, years of practice, conference attendance, qualification and gender—indicating partial confounding by correlated covariates. Conference attendance was the strongest independent predictor of motor‐learning use (aOR 3.72, 95% CI 1.44–9.58, *p* = 0.007) and was significantly associated with high EBP frequency (aOR 2.59, 95% CI 1.01–6.62, *p* = 0.048).

**Table 5:** Multivariable logistic regression models for three prespecified practice outcomes (*N* = 118). Adjusted odds ratios (aORs) with 95% confidence intervals.

Predictor	Any EBP challenge (*y* *e* *s* = 1)	High EBP frequency (≥ ‘most of the time’)	Motor‐learning use (*y* *e* *s* = 1)
Private (vs. Governmental) sector	2.51 [0.90–7.01] *p* = 0.078	0.72 [0.29–1.77] *p* = 0.474	0.78 [0.31–1.93] *p* = 0.587
Years of practice (per year)	0.96 [0.90–1.03] *p* = 0.273	1.07 [1.00–1.14] *p* = 0.060	1.00 [0.94–1.06] *p* = 0.923
GCC (vs. non‐GCC)	0.54 [0.16–1.80] *p* = 0.317	0.71 [0.26–1.94] *p* = 0.506	1.46 [0.53–4.04] *p* = 0.470
Conference attendance (Yes)	1.85 [0.66–5.18] *p* = 0.241	2.59 [1.01–6.62] *p* = 0.048	3.72 [1.44–9.58] *p* = 0.007
Postgraduate (vs. Bachelor)	1.08 [0.31–3.73] *p* = 0.906	0.83 [0.27–2.56] *p* = 0.747	1.54 [0.48–4.94] *p* = 0.464
Female (vs. Male)	1.18 [0.48–2.89] *p* = 0.725	0.93 [0.40–2.14] *p* = 0.865	—
Model likelihood ratio	*χ* ^2^ = 9.51, *p* = 0.147	*χ* ^2^ = 11.28, *p* = 0.080	*χ* ^2^ = 11.50, *p* = 0.042

*Note:* All models adjusted for the covariates listed. The motor‐learning model omits gender. A separate unadjusted gender × NDT‐use test (*χ* ^2^(1) = 4.78, *p* = 0.029, OR = 2.67 [1.18–6.06]) is reported in Section 3.3.

Internal consistency of the six‐item EBP‐barriers inventory was modest: Cronbach *α* was 0.40 among the 85 respondents who endorsed any barrier and 0.48 in the full sample. Item–total correlations ranged from −0.11 to 0.36, indicating that the six barriers are best treated as distinct constructs rather than as a unidimensional scale. This is an important finding in its own right: the six barrier items function as independent indicators, and future regional research should consider expanded, multi‐item subscales (e.g., Upton′s EBPQ [37] or adapted versions for Arabic‐speaking contexts) rather than aggregating dichotomous barrier endorsements into a composite score.

*Placeholder Text*Click or tap here to enter text.Click or tap here to enter text.Click or tap here to enter text.*Placeholder Text*

## 4. Discussion

This exploratory multicountry survey provides one of the first empirical descriptions of neurology and cognitive OT practice across the MENA region, drawing on 118 licensed occupational therapists from 13 countries. Five findings warrant particular attention and are discussed in turn: (a) an apparent evidence‐practice gap in the dominance of neurodevelopmental treatment; (b) the tension between occupation‐focused framing at the level of models and impairment‐focused assessment in the same respondents; (c) a structurally patterned and subregionally differentiated profile of EBP barriers; (d) the role of continuing professional development in the motor‐learning‐use signal; and (e) sample‐composition factors that must shape interpretation.

### 4.1. Why Neurodevelopmental Treatment Dominates: Closer Look at the Evidence‐Practice Gap

The 71.2% reported use of neurodevelopmental treatment (NDT/Bobath) is the single most striking finding. Contemporary systematic reviews in adult stroke rehabilitation consistently find NDT is not superior—and is often inferior—to task‐specific and motor‐learning approaches for both upper‐limb and lower‐limb activity outcomes [38, 39]. Major adult stroke clinical practice guidelines, including the American Heart Association/American Stroke Association guideline [40], the Canadian Stroke Best Practices [41], the UK National Clinical Guideline for Stroke [42] and NICE NG236 [43], recommend task‐oriented repetitive practice as first‐line therapy and do not endorse NDT. Against this evidence base, more than 70% reported use of NDT among MENA OTs suggests a substantial evidence‐practice gap. The strength of this inference is moderated by the survey′s measurement of reported, rather than observed, practice (see Sections 4.1 and 5): respondents endorsing NDT use may include those who use the approach predominantly, those who use it alongside other approaches and those who identify with it as a professional tradition without applying it as the dominant intervention in daily clinical minutes. The 71.2% figure should therefore be read as evidence of widespread reported affiliation with NDT rather than as a precise estimate of the proportion of clinical time devoted to NDT in the region.

Three explanations plausibly contribute, none mutually exclusive and all testable in follow‐up work. First, NDT has been the dominant continuing education offering in the MENA region for three decades. Bobath and NDT instructor courses are well established in the GCC, the Levant and Iran, often with returning cohorts and structured mentorship, whereas task‐specific training, constraint‐induced movement therapy, and mirror therapy are more recent arrivals with patchier regional course availability. A training‐supply hypothesis would predict that reported NDT use reflects both genuine practice and the depth of accumulated continuing‐professional‐development exposure. Second, the survey instrument captured reported use rather than observed use—a critical distinction. Respondents who selected NDT may be reporting what they learned, what they identify with professionally, or what they use some of the time alongside other approaches, rather than the intervention dominating their daily clinical minutes. Observational work in stroke rehabilitation has documented gaps of this kind between therapists′ stated approach and their enacted practice—for example, physiotherapists who described their practice as eclectic but whose treatment content followed a traditional Bobath model, and interventions where self‐reported use of an approach exceeded its observed delivery [44, 45]. The 63.6% reported use of motor‐learning approaches alongside the 71.2% reported NDT use is consistent with this interpretation: most respondents are reporting multiple coexisting orientations, not mutually exclusive single approaches. Third, NDT use differed by gender (79.2% female vs. 58.7% male; OR 2.67, 95% CI 1.18–6.06, *p* = 0.029). The substantive meaning of this gender difference is uncertain in a cross‐sectional, single‐region sample, but it warrants explicit note because gender composition and workforce demographics differ across the sampled countries [17, 46]; the finding is hypothesis‐generating rather than a causal claim.

The strongly adjusted association between conference attendance and motor‐learning use (aOR 3.72, 95% CI 1.44–9.58, *p* = 0.007) is not evidence that continuing education causes practice change, but it is consistent with the hypothesis that structured exposure to contemporary stroke‐rehabilitation evidence changes what therapists report doing. Future regional research must distinguish reported from enacted practice—ideally through chart review, observation or coded video—because self‐reported model endorsement is insufficient for implementation research.

### 4.2. Occupation‐Focused Models Alongside Impairment‐Focused Assessment

A second tension emerges between model and assessment profiles. PEO and CMOP‐E are person‐and‐environment models that centre occupational performance; respondents who endorsed them also reported broad attention to social participation (64.4%), work (60.2%), IADL (58.5%), education (50.8%) and leisure (50.0%) as intervention areas—a profile consistent with contemporary occupation‐focused practice. Yet the assessment profile is heavily weighted toward impairment‐level tools (Range of Motion 54.2%, Manual Muscle Test 50.8%, Ashworth 47.5%, grip/pinch 37.3%), with occupation‐focused (COPM 49.2%) and cognitive‐functional instruments (FIM 65.3%, MoCA 61.9%, MMSE 52.5%) competing for dominance.

This is not necessarily contradictory. In neurological practice, impairment‐level measurement is often clinically essential alongside performance‐level measurement. The finding does, however, raise two regionally specific concerns. First, no peer‐reviewed Arabic psychometric validation of the FIM was identified in a structured literature scan for this manuscript, and Arabic MoCA validation, while expanding [47–51], is sparse in stroke‐specific populations, and available normative work indicates that performance is affected by education level, supporting the use of education‐adjusted cut‐offs in MENA samples [49, 51]. Second, the continuing prominence of FIM use sits awkwardly against its 2019 removal from the United States CMS IRF‐PAI in favour of the Section GG CARE Item Set—a development with implications for regional training and benchmarking. Arabic‐language adaptation and validation of core neurological OT outcome measures (FIM, Barthel, LOTCA, stroke‐specific MoCA) is a concrete, measurable regional research priority.

### 4.3. A Structurally and Subregionally Patterned Barrier Profile

Respondents most commonly reported organisational rather than individual EBP barriers, replicating a pattern seen among Jordanian occupational therapists [52], Kuwaiti paediatric occupational therapists [16], Saudi OT practitioners [53], and the broader international allied‐health literature [54]. At the aggregate level, this suggests that regional EBP promotion is unlikely to succeed through individual‐level upskilling alone; structural levers—protected time, institutional access to databases and guidelines, evidence mentorship and accountable EBP governance—are necessary. The stratified analysis (Table 4) reveals a more textured picture not visible at the aggregate level.

#### 4.3.1. Qualification‐Linked: A Counter Intuitive Postgraduate Signal

Postgraduate‐qualified respondents (master′s, OTD, PhD; combined *n* = 26) reported significantly higher rates of the organisational‐support barrier (73.1% vs. 45.7%, *p* = 0.025) than bachelor‐level colleagues, with a similar nonsignificant trend for time to search evidence (65.4% vs. 43.5%, *p* = 0.080). This pattern—more training, more barriers reported—may seem paradoxical but is consistent with EBP‐development models in which increasing research literacy raises the floor of what one considers ‘sufficient’ evidence engagement, rendering structural gaps in database access and protected time more visible [37, 54]. From a policy standpoint, this signal is important: investments in postgraduate OT pathways across MENA cannot be expected to reduce perceived EBP barriers in isolation; they must be accompanied by institutional infrastructure that enables postgraduates to translate their training into practice.

#### 4.3.2. Sector‐Linked: Confidence in Research Appraisal

The work‐sector difference was concentrated in a single item: low confidence in appraising research, reported by 32.7% of private‐sector respondents versus 11.6% of governmental‐sector respondents (*p* = 0.010). Private‐sector respondents also reported a greater number of distinct barriers overall (mean 2.63 vs. 1.87; Cohen *d* = 0.54, medium effect), and the unadjusted any‐challenge OR was 2.91 (95% CI 1.18–7.18, Fisher *p* = 0.022). Plausible structural mechanisms include less embedded in‐service training, fewer postgraduate‐trained colleagues in smaller private clinics, less structured access to library and database subscriptions and market pressures that compress direct‐service time. This is compatible with the broader MENA health‐workforce literature [55–57] and is a priority hypothesis for confirmatory implementation research. In adjusted models (Table 5), the private‐sector signal for the any‐challenge outcome attenuated to borderline (aOR 2.51, 95% CI 0.90–7.01, *p* = 0.078), indicating partial confounding by correlated covariates rather than a fully independent sector mechanism.

#### 4.3.3. Subregional Patterning: The Largest Exploratory Signal

The largest pattern in the stratified analyses was subregional, and we describe it next while noting the limits of inference it can support. Respondents practising outside the GCC (combined Levant + Maghreb + non − Arab MENAT; *n* = 37) reported three‐ to four‐fold higher rates of confidence in appraising research (37.8% vs. 12.3%, OR 4.32, 95% CI 1.69–11.04, *p* = 0.003), confidence in implementing research (35.1% vs. 12.3%, *p* = 0.008) and poor evidence quality (54.1% vs. 25.9%, *p* = 0.006) than GCC respondents. Organisational and time‐related barriers, by contrast, did not differ by GCC versus non‐GCC subregion. This suggests that the kinds of barriers dominating EBP experience differ systematically by regional health‐system context. A descriptive four‐way breakdown shows that the Maghreb (Morocco, *n* = 8) sits between the GCC and Levant on most barriers but shares the Levantine pattern of elevated poor‐evidence‐quality endorsement, whereas the small non‐Arab MENAT subsample (Iran + Turkey, *n* = 5) reports the highest rates across all six items—a descriptive signal that warrants targeted country‐specific follow‐up but cannot be subjected to formal inference at this sample size.

Several country‐level factors could in principle contribute to the observed subregional difference, although the present data do not test any of them directly and the four‐way subregional sample sizes preclude inference at the country level. Candidate hypotheses for future, adequately powered research include (i) differences in library and database infrastructure (many GCC institutions, with oil‐revenue‐supported health‐sciences universities, have wide access to international journals and evidence databases; Levantine, Iranian and some North African institutions often operate on more constrained budgets, further stressed in Lebanon and Syria by extended economic and political crises [55]); (ii) differences in language of clinical training and clinical documentation (Farsi, Turkish and French clinical‐education systems create additional translation steps for primarily English‐language evidence—a particularly relevant consideration for the Maghreb, where French‐medium clinical education predominates [27]); (iii) differences in regulatory frameworks and statutory continuing education requirements (the Saudi Commission for Health Specialties and the Kuwait Ministry of Health mandate continuous medical education credits tied to licensure, whereas many other regional regulators have weaker CME infrastructures); and (iv) differences in postgraduate neuro‐OT training availability (Iran, Turkey and Jordan have the longest‐established postgraduate OT programmes in the sample, whereas the GCC lacks neuro‐OT‐specific postgraduate pathways apart from general OT master’s tracks, creating different kinds of workforce‐to‐evidence friction). Each of these is a candidate hypothesis rather than an interpretation supported by the present data: the four‐way subregional sample sizes (with 10 of 13 countries each contributing fewer than 15 respondents) are too small to test any country‐level mechanism, and the heterogeneity of the combined non‐GCC stratum further constrains the inferences that can be drawn. The subregional difference is therefore best interpreted as an exploratory empirical signal that future country‐specific and multisite regional research, adequately powered and designed to measure these candidate factors directly, may help to explain.

### 4.4. Continuing Professional Development as a Regionally Actionable Lever

Conference attendance was associated with reported use of motor‐learning approaches even after adjustment for sector, qualification, subregion and years of practice (aOR 3.72, 95% CI 1.44–9.58, *p* = 0.007), and with higher EBP‐use frequency (aOR 2.59, 95% CI 1.01–6.62, *p* = 0.048). These associations are cross‐sectional and should not be read as evidence that conference attendance causes practice change. Several alternative explanations are equally compatible with the data: practitioners who already favour motor‐learning approaches may be more likely to attend conferences (reverse causation); conference attendance may serve as a marker for underlying motivation, English‐language proficiency, institutional permission and funding, or research engagement that themselves drive practice; and unmeasured confounders may explain the observed association. Continuing professional development remains a reasonable practical focus for regional workforce planning, but the strength of the present association should not be taken as direct evidence of its causal efficacy. In the regional ecosystem, the AOTRG—a WFOT regional member since 2012 [29]—and national associations are well placed to coordinate periodic workforce‐relevant conference content, with explicit emphasis on task‐specific training, constraint‐induced movement therapy, mirror therapy and other motor‐learning‐aligned approaches endorsed by current CPGs.

### 4.5. Sample Composition and How It Shapes the Profile

Four sample‐composition features shape every finding reported here, and this study′s interpretation must be tempered accordingly. In addition, the post hoc stratified analyses reported in Table 4 were not adjusted for multiple comparisons and rest on subgroup cells that are in several cases small (e.g., *n* = 26 postgraduate respondents; *n* = 5 non‐Arab MENAT respondents); these analyses should therefore be read as exploratory signals to be confirmed in larger, country‐specific samples rather than as established subgroup effects. First, 54.2% of respondents had 1–5 years of clinical experience and 56.8% were aged 20–29. This early‐career skew implies that the reported practice profile reflects comparatively recent training exposure. Newer graduates are more likely to have been exposed to motor‐learning literature and occupation‐based models during preprofessional education, whereas long‐standing NDT‐trained practitioners—especially those in practice before the 2010s task‐specific‐training evidence base matured—may be under‐represented. The 71.2% reported NDT use in an early‐career‐skewed sample should therefore be understood as a conservative lower‐bound estimate for the regional workforce, if anything.

Second, the three‐country concentration (Kuwait 37.3%, Saudi Arabia 19.5%, Jordan 11.9%—together 68.6%) means that overall profile descriptions are weighted toward the practice contexts of three specific health systems. The aggregate figures describe a respondent sample dominated by Kuwait, Saudi Arabia and Jordan, not a representative regional average; readers should interpret them accordingly. The Maghreb (Morocco, *n* = 8) and non‐Arab MENAT (Iran/Turkey, *n* = 5) subsamples are particularly underpowered and contribute meaningfully only to the descriptive profile and to subregion‐stratified comparisons grouping these strata with the Levant.

Third, female respondents constituted 61.0% of the sample, which is broadly consistent with international OT workforce literature [17]. Nonetheless, gender‐by‐country composition was not uniform, and the gender × NDT‐use association (OR 2.67; *p* = 0.029) should be interpreted against the possibility of differential uptake of particular continuing‐education pathways by gender in different countries—a hypothesis that deserves formal country‐stratified examination in larger samples.

Fourth, the 77.1% conference‐attendance rate in this sample almost certainly exceeds the conference‐attendance rate of the broader regional workforce. Online‐survey respondents who find their way to professional‐network recruitment are more likely to be professionally engaged and EBP‐oriented than the regional workforce average. This self‐selection is structurally built into convenience and snowball sampling [31] and imposes an upper bound on how far aggregate EBP‐frequency statistics can be generalised. It also implies that the barriers reported here are the barriers experienced by therapists who are already comparatively engaged with professional networks; barriers reported by less‐engaged practitioners, who are under‐represented in the sample, are likely to be different in profile and possibly greater in magnitude.

### 4.6. Alignment With WHO Rehabilitation 2030 and Regional Policy

The findings align with the WHO Rehabilitation 2030 workforce pillar [22] and with the 2023 WHA76.6 resolution on strengthening rehabilitation in health systems [23]. The WHO Package of Interventions for Rehabilitation [58] explicitly includes stroke, traumatic brain injury, Parkinson disease, and dementia—all represented in this workforce profile—and the WHO Rehabilitation Competency Framework [59] provides a structure against which regional continuing‐education curricula can be benchmarked. WFOT minimum education standards [60] are unevenly applied across the 13 surveyed countries, and postgraduate neuro‐OT pathways remain concentrated in a minority (notably Iran, Turkey and Jordan). The 78.0% bachelor‐level preparation in our sample, together with the conference‐attendance signal, indicates that scalable continuing‐education infrastructure and regional postgraduate pathways are actionable priorities.

### 4.7. Clinical, Educational and Policy Implications

Practically, five implications follow. First, regional continuing‐education programmes should explicitly teach and model task‐specific, motor‐learning and constraint‐induced movement therapy approaches for adult stroke rehabilitation, recognising their superior evidence base over NDT [38–43]. Second, institutions—public and private—should invest in protected EBP time, database access and evidence‐mentorship structures, because the barriers identified are structural and responsive to organisational design. Third, postgraduate OT pathway expansion must be accompanied by matched infrastructure investment; otherwise it may simply make structural EBP barriers more visible without enabling practitioners to cross them. Fourth, Arabic and French‐language validation of core neurological OT assessments (particularly the FIM, Barthel, LOTCA and stroke‐specific MoCA) should be a coordinated regional priority, with Maghreb countries especially well placed to lead the French‐language adaptation work. Fifth, national regulators and WFOT regional governance—through the AOTRG—should support periodic workforce monitoring, regional evidence synthesis and country‐specific neuro‐OT clinical practice guidelines.

## 5. Strengths and Limitations

To the author′s knowledge, this is the first multicountry survey to describe neurology and cognitive OT practice across 13 MENA countries with formal STROBE and CHERRIES reporting, use of Wilson 95% confidence intervals throughout, and a prespecified multivariable analysis plan including aORs and stratified barrier comparisons. These methodological choices strengthen the interpretability of the descriptive profile relative to earlier regional OT surveys.

Several limitations must be acknowledged. First, the convenience and snowball sampling design and open‐link distribution mean that the 118 analysed respondents are unlikely to be representative of the region′s occupational‐therapy workforce; Kuwait, Saudi Arabia and Jordan together contributed 68.6% of responses, and seven countries provided fewer than five responses each. Findings are best understood as a respondent‐sample snapshot, not a regional estimate. Second, the open‐link distribution means the total invited population is unknown, a conventional response rate cannot be computed, and self‐selection bias toward more professionally engaged and EBP‐oriented therapists is plausible (see Section 4.5). Third, self‐report through a structured online instrument is subject to recall and social‐desirability biases; reported model, frame‐of‐reference and assessment use may reflect familiarity and training history more than day‐to‐day enacted practice—a distinction the present design cannot resolve and that future work should address through observation, chart review or coded video [44]. Fourth, the instrument was developed specifically for this study. Although content validity was supported by a five‐member expert panel following the Polit–Beck procedure [32, 33] and the instrument was pilot‐tested with 30 occupational therapists, formal psychometric evaluation of total scores was not undertaken. The six‐item EBP‐barrier inventory showed limited internal consistency (*α* ≈ 0.40–0.48), indicating that the six barriers are better treated as distinct indicators than as a summated scale. Fifth, the monthly‐income variable was collected with inconsistently ordered response labels in the deployed form; income was therefore reported descriptively but not analysed further. Sixth, translation and linguistic equivalence across Arabic, English, French, Turkish and Farsi settings were not formally assessed using a forward–back translation protocol [61]; findings across language contexts must be interpreted with that caveat. Seventh, the cross‐sectional design precludes causal inference: all associations reported—including the subregional, private‐sector and conference‐attendance signals—are correlational and hypothesis‐generating. Eighth, the Maghreb (*n* = 8) and non‐Arab MENAT (*n* = 5) subsamples are too small to support stable inference, and findings stratified by these specific subregions are descriptive only.

## 6. Conclusion

This exploratory multicountry cross‐sectional survey provides an initial empirical profile of neurology and cognitive OT practice across 13 MENA countries. Respondents were predominantly early‐career, bachelor‐level, female practitioners working in governmental or outpatient settings, with stroke as the dominant clinical focus. The reported practice profile reflects both occupation‐focused framing (PEO, CMOP‐E, ADL/IADL/social participation intervention) and a persistent evidence‐practice gap in favour of neurodevelopmental treatment relative to contemporary motor‐learning and task‐specific recommendations. Exploratory stratified analyses identified candidate structural and subregional patterning of perceived EBP barriers, with larger confidence‐ and evidence‐quality‐related barriers reported outside the GCC and counter‐intuitively higher organisational‐support barriers reported by postgraduate‐qualified respondents; these patterns require confirmation in larger, country‐specific samples. Conference attendance was associated with reported use of motor‐learning approaches, although this cross‐sectional association may reflect reverse causation or unmeasured confounders rather than a direct effect of conferences on practice. Findings identify concrete, actionable priorities for regional continuing education, organisational EBP support, Arabic and French outcome‐measure adaptation, postgraduate pathway investment and coordinated workforce monitoring—priorities aligned with the WHO Rehabilitation 2030 workforce agenda and readily taken forward through the AOTRG. Country‐specific and multicentre confirmatory research using representative sampling, longitudinal designs and observational rather than self‐report measurement of practice is now needed.

## Author Contributions

**Hamad AlHamad:** conceptualization, methodology, validation, formal analysis, investigation, data curation, writing – original draft, writing – review and editing, project administration, funding acquisition.

## Funding

This study was supported by the Kuwait University, 10.13039/501100004482, ZN06/22.

## Ethics Statement

Ethical approval was obtained from the Kuwait University Ethical Review Board (Approval No. 312). The study was conducted in accordance with the Declaration of Helsinki. Electronic informed consent was obtained from all participants

## Conflicts of Interest

The author declares no competing interests.

## Data Availability

The data that support the findings of this study are available from the corresponding author upon reasonable request.
